# Benefits of Residual Aluminum Oxide for Sand Blasting Titanium Dental Implants: Osseointegration and Bactericidal Effects

**DOI:** 10.3390/ma15010178

**Published:** 2021-12-27

**Authors:** Javier Gil, Román Pérez, Mariano Herrero-Climent, Maria Rizo-Gorrita, Daniel Torres-Lagares, Jose Luis Gutierrez

**Affiliations:** 1 Bioengineering Institute of Technology, Faculty of Medicine and Health Sciences, International University of Cataluña, c. Josep Trueta s/n. Sant Cugat del Valles, 08125 Barcelona, Spain; rperezan@uic.es; 2 Faculty of Denstistry, International University of Cataluña, c. Josep Trueta s/n. Sant Cugat del Valles, 08125 Barcelona, Spain; 3 Porto Dental Institute, 4150-518 Porto, Portugal; dr.herrero@herrerocliment.com; 4 Faculty of Dentistry, University of Seville, C/Avicena s/n., 41009 Seville, Spain; mrizog@us.es (M.R.-G.); danieltl@us.es (D.T.-L.); jlpg@us.es (J.L.G.)

**Keywords:** titanium implants, osseointegration, surfaces, alumina

## Abstract

Objectives. The purpose of this work was to determine the influence of residual alumina after sand blasting treatment in titanium dental implants. This paper studied the effect of alumina on physico-chemical surface properties, such as: surface wettability, surface energy. Osseointegration and bacteria adhesion were determined in order to determine the effect of the abrasive particles. Materials and Methods. Three surfaces were studied: (1) as-received, (2) rough surface with residual alumina from sand blasting on the surface and (3) with the same roughness but without residual alumina. Roughness was determined by white light interferometer microscopy. Surface wettability was evaluated with a contact angle video-based system and the surface free energy by means of Owens and Wendt equation. Scanning electron microscopy equipped with microanalysis was used to study the morphology and determine the chemical composition of the surfaces. Bacteria (Lactobacillus salivarius and Streptococcus sanguinis) were cultured in each surface. In total, 110 dental implants were placed into the bone of eight minipigs in order to compare the osseointegration. The percentage of bone-to-implant contact was determined after 4 and 6 weeks of implantation with histometric analysis. Results. The surfaces with residual alumina presented a lower surface free energy than clean surfaces. The in vivo studies demonstrated that the residual alumina accelerated bone tissue growth at different implantation times, in relation to clean dental implants. In addition, residual alumina showed a bactericidal effect by decreasing the quantity of bacteria adhering to the titanium. Conclusions. It is possible to verify the benefits that the alumina (percentages around 8% in weight) produces on the surface of titanium dental implants. Clinical relevance. Clinicians should be aware of the benefits of sand-blasted alumina due to the physico-chemical surface changes demonstrated in in vivo tests.

## 1. Introduction

Sand blasting or grit blasting is a widely used surface treatment method in order to achieve the appropriate roughness of the titanium dental implants in relation to the biological response. Alumina particles (Al_2_O_3_) are the most commonly used for this purpose, followed by an acid etching treatment. Sand blasting treatment produces a macroroughness with very abrupt peaks and valleys, and it is the acid treatment that rounds the structure and causes a micro-roughness within the macroroughness exerted by the abrasive projection. It is well known that acid treatment alone does not produce the right roughness for biological osseointegration. Sand blasting is the key treatment to obtain a favorable titanium surface [1,2,3]. Furthermore, many types of abrasives have been used, but the particles must be harder than titanium to produce roughness, and this fact means that materials, such as titanium oxides, calcium phosphates, among others, cannot be successfully applied as they do not obtain the appropriate roughness [4,5,6]. Alumina is the most commonly used abrasive by commercial companies and most implants are treated with these particles, as they give excellent osseointegration results and in no case have foreign body reactions or allergic effects. However, it is not only the nature of the abrasive that has an effect, but also the projection parameters, such as the distance from the projection gun to the titanium surface, the projection pressure, the saturation time, projection diameter, etc. In addition, there are other parameters of the alumina that will influence the roughness, such as the size or shape of the particles.

The sand blasting produces a roughness, the value of which depends on the size, nature of abrasive particles, projection pressure and the distance from the gun to the titanium surface. The roughness, ranging from 1 to 3 μm, is considered as an optimal surface for osseointegration. A strong and rapid osseointegration is considered an important key for the success of titanium dental implants [1,2,3,4,5].

Other techniques have been used to roughen the surfaces of dental implants, one of the most recent being thermal spray processing (TPS), which is sometimes combined with sand blasting and etching-acid treatments. This treatment produces roughness between 1.5 to 2 μm, which favors osseointegration [6,7].

Roughness plays an important role in osteoblast adhesion, proliferation and differentiation. In addition, bacterial adhesion to the rough surface must be taken into account. Therefore, we have to take into account the cells and bacteria to obtain the optimal surface [8,9].

Furthermore, the sand blasting produces a compressive residual stress on the surface, which produces an increase in the mechanical properties, especially in the fatigue life [6,7,8]. The projection at a high pressure of abrasives produces an important increase in the compressive residual stress on the surface. This compression hinders crack nucleation by fatigue on the surface, being the crack nucleation on the matrix of around 10 μm from the surface. This fact increases the number of cycles to nucleate the crack, produced by mechanical cycles which produce an important improvement in the fatigue life and provide the dental implant with an excellent long-term mechanical behavior [10,11,12,13,14,15,16,17].

Many authors have found other important parameters, which play roles in the rate and quality of osseointegration: chemical composition, wettability, surface energy and zeta potential, among others [18,19]. Sand blasting treatment increases the contact angles. As a consequence, these implants have presented with more hydrophilic behavior. The results of surface energy of the sand blasting implants, comparing dispersive or polar components of surface energy, showed that there was a general trend in the polar component decreasing when the samples were treated by sand blasting. The polar component is important, as it can facilitate the adsorption of human osteoblast precursor proteins due to the negative charge density on the surface [20,21]. An important requirement for the bone regeneration on titanium dental implant is the cleanliness, and contamination could avoid the osseointegration. In the literature, this aspect has not been studied in depth and controversial opinions can be found [22,23,24,25,26].

Moreover, the abrasive particles could favor the microbial contamination and in consequence these could compromise the stability of dental implants, increasing the risk of inflammatory reactions in the surrounding soft tissues. Several clinical tests [27,28] studied the presence of different bacteria surrounding peri-implant soft tissues, showing that treatment with 0.20% chlorhexidine improves the soft and hard peri-implant tissue healing. These studies results have helped to select the bacteria to be considered for our in vitro tests. The present study did not verify the bactericidal effect of the materials because further studies should be carried out, although bacterial colony counts can give an idea of the character of the material.

The objective of this study was to assess the in vitro and in vivo behavior of three different surfaces (as-received and two sand blasting with alumina: a batch with residual alumina and another without alumina, due to the cleaning process) and analyze the effect of the residual alumina on the surface in osseointegration and bactericide behavior.

## 2. Materials and Methods

### 2.1. Samples and Treatments

Ninety cylindrical samples (5 mm diameter, 2 mm width) were cut from a bar of commercially pure titanium (cp-Ti) grade 3, with a chemical composition and mechanical properties compliant with the standard ASTM F67:2006.

Three different surface conditions were studied:

- (Ctr). As-received lathed cut titanium samples (control samples).
- (Al_2_O_3_). Shot-blasted surfaces: The samples were shot-blasted with Al_2_O_3_ particles with a size range of 212–300 μm at a pressure of 2.5 MPa until saturation. After the blasted process, all specimens were washed with distilled water, ethylic alcohol and acetone, sonicated in ultra-pure water, dried at room temperature, packaged and autoclaved at 121 °C for 30 min.
- (Clean). Shot-blasted surfaces: The specimens were projected with alumina at the same pressure of 2.5 MPa. The abrasive particles had the same size of the (Al_2_O_3_) group. After the shot-blasted process, a special cleaning was performed. Samples were ultra-sonicated in acetone at high pressure for 5 h. These samples were analyzed by EDX microanalysis in order to ensure the absence of alumina particles. The sensibility of the microanalysis was around 0.8%.

### 2.2. Implants

**Figure 1:**
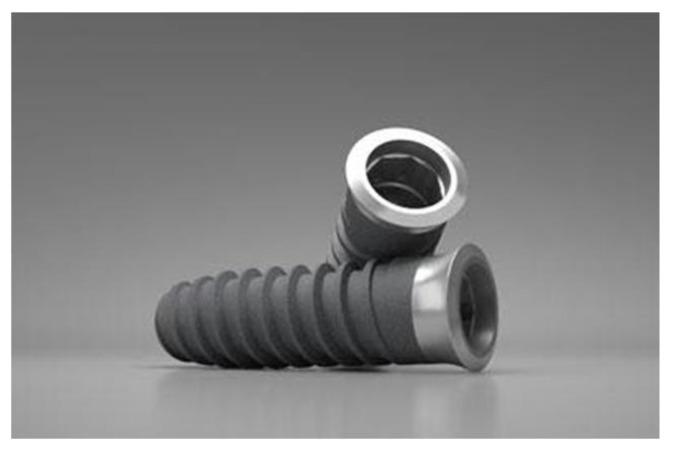
Essential implants by Klockner used in the research.

The screw-shaped dental implants (Essential by Klockner, Soadco Co, Escaldes Engordany, Andorra) were 3.8 mm in diameter and 12.0 mm in length with 1.0-mm pitch and 1.5-mm long collar (batch series 0032-35789A309) (Figure 1). The dental implants used were machined from the same batch of cp-Ti (grade 3) bar was used to fabricate the disks. One hundred and ten dental implants were implanted for the in vivo study.

### 2.3. Surface Roughness and Topography

Roughness was evaluated by means of white light interferometer microscopy (Wyko NT1100, Veeco, Plainview, NY, USA). The surface analysis area was 459.9 · 604.4 μm^2^ for each measurement for all studied surfaces. Seven measurements were realized for each sample. The analysis of the results was completed by Wyko Vision 232TM software (Veeco, Plainview, NY, USA). The measurements of Sa and Sm were made in three different surfaces of each type of surface treatment. Sa is defined as the arithmetic average of the absolute values of the distance of all points of the profile to the mean line. Sm is the mean spacing between peaks. Finally, the index area is the ratio between the real surface area and the nominal surface area.

The qualitative analysis of the topography was realized by means of a scanning electron microscope (SEM) (JSM 6400, Jeol, Tokyo, Japan).

Surface wettability was evaluated with a contact angle video-based system (Contact Angle System OCA15plus, Dataphysics, Filderstadt, Germany) and analyzed with proprietary software (SCA20, Dataphysics Filderstadt,Germany). All the tests were realized at a constant temperature and 100% relative humidity. Static contact angles, CA, of three reference liquids (ultrapure distilled water (MilliQ), di-iodomethane and formamide) were measured by the sessile drop method. 2 μL drops were dispensed on the substrate surface under a controlled temperature (T = 25 °C) and 100% relative humidity. The wettability was studied with the help of a contact angle goniometer (OCA 15+, Dataphysics, Filderstadt, Germany).

Dispersion and polar components and the total surface free energy (SFE) for all series were calculated. CA was measured with two different liquids on each material: ultrapure distilled water (MilliQ) and di-iodomethane [29,30,31]. The SFE and its components were obtained by means of Owens and Wendt equation [30,32,33].

(1) $$\gamma_{S}=\gamma_{S}^{d}+\gamma_{S}^{p}$$

(2) $$\gamma_{L}\cdot (1+\cos\theta )=2\cdot ({\left(\gamma_{L}^{d}\cdot \gamma_{S}^{d}\right)}^{1/2}+{\left(\gamma_{L}^{p}\cdot \gamma_{S}^{p}\right)}^{1/2})$$

where $\gamma^{d}$ is due to the interactions arising from induced and dipole-dipole forces (London or ‘dispersion’), and $\gamma^{p}$ is the ‘polar’ component arising from the interaction between permanent dipoles.

### 2.4. Bacterial Strains and Growth Conditions

The bacteria strains *S. sanguinis* CECT 480 and *L. salivarius* (CECT 4063) (Colección Española de Cultivos Tipo, Valencia, Spain) were used in this study. Bacteria were cultured in Todd-Hewitt broth at 37 °C in a 5% CO_2_-enriched atmosphere.

Surface energy of the bacteria was determined according to an adaptation of the microbial adhesion to solvents test (MATS) [34]. Besides, this method is a good method in order to quantify the bacteria adhesion on titanium surfaces [35]. Bacteria were picked by centrifugation at 4500 g for 15 min at 4 °C, and washed with phosphate buffered saline (PBS) 0.15 M. The bacteria quantification was realized by optical density measured at 550 nm (A_0_). The liquids used as solvents, according to the MATS test were: hexane, diethyl ether and chloroform. After that, 3 mL of bacteria suspension was placed in 9 tubes and 400 μL of solvent was added (3 samples for each solvent), incubated at 22 °C for 10 min and mixed on a vortex for 60 s. The aqueous phase was extracted after 15 min and its optical density measured at 550 nm (A_1_). The average of microbial adhesion to solvent was calculated as: (1 − A_1_/A_0_) × 100.

Samples were cleaned with 70% ethylic alcohol, acetone and distilled water, dried at room temperature and sterilized in autoclave. The quantification of bacteria adhered was realized using: Streptococcus sanguinis (CETC 480, Valencia, Spain) and Lactobacillus salivarius (CECT 4063, Valencia, Spain), very usual in the mouth. C.p-Ti disks with 5 mm diameter and 2 mm thickness were used in this study. Treated samples were placed into 24-well plates and incubated with 1 mL of bacterial suspensions (1 × 10^8^ CFU/mL) for 2 h at 37 °C. After this time, the medium was aspired and samples washed twice with PBS. Adherent bacteria were detached by vortexing the disks for 5 min in 1 mL of PBS. These bacteria were then seeded using serial dilutions on TH agar plates for *S. sanguinis*, MRS agar plates for *L. salivarius* and tryptic soy agar for oral plaque. The plates were then incubated at 37 °C for 24 h and the resulting colonies were counted.

### 2.5. In Vivo Test

**Figure 2:**
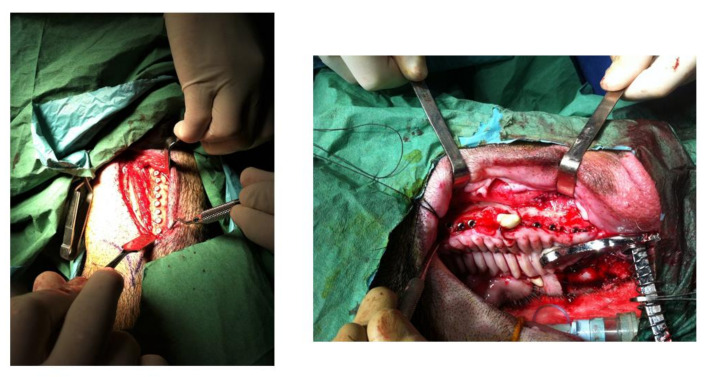
Insertion of the dental implants in the mandibular bone.

Each animal received 16 implants: 4 for control, 6 for Al_2_O_3_ and 6 for clean. Eight implants were placed in each hemi-arcade of the mandibular bone. Four animals for each time implant placement received dental implants and were sacrificed after 4 and 6 weeks after surgery. In Figure 2, the implantation in the bone can be observed.

Eight 12-year-old female minipigs were used in the in vivo studies after ensuring that the bone available to these animals was mature. The surgeries were performed according to the protocol approved for this study by the Faculty of Veterinary Sciences of the University of Cordoba (Spain) under the registration number P01/0144. The minipigs were fed a soft diet until slaughter. Oral prophylaxis was performed 3 weeks prior to surgery in an aseptic manner.

Four months prior to dental implant placement, single and multi-rooted extractions of all teeth of each minipig were performed. After the extractions, the hemorrhages were easily corrected and no severe swellings were observed, which resolved within a short period of time. Radiographic images of the mandible and maxilla of each minipig were taken 24 h before the surgeries to avoid the presence of root canals, and to design the placement of the dental implants.

The animals were not fed one day before surgery. The minipigs were pre-anesthetized with xylazine and ketamine and maintained under gaseous anesthesia (5% isoflurane/O_2_). The minipigs were hydrated by infusion of lactated Ringer’s solution. Anesthesia was monitored throughout by measuring heart rate, power and respiration rate of the animals. The animals were picked at a certain frequency to check the corneal reflex [30,31].

Dental implants were placed in the mandible and maxilla of the animals according to the drilling technique proposed by the implant manufacturer. After surgery, the implant collars were exposed and held at gingival level. After placing the implants in the bone bed, the flap margins were adapted and sutured without tension using bioresorbable polyglycine 910-Vycril© 3-0, Ethicon, Raritan, NJ, USA.

After surgery and in order to avoid pain, buprenorphine HCl and amoxicillin were administered to prevent infection. The animals were observed several times a day for possible problems, especially inflammation, dehiscence and infection.

After animal sacrifice, 16 mm thick sections including implants, alveolar bone and surrounding mucosa were cut with a diamond saw irrigated with tissue elasticity change control and titanium to avoid fractures in the specimens (Accutom 50, Struers, Cleveland, OH, USA) and were radiographed. The specimens were cleaned in sterile saline and placed in 10% buffered formalin solution [36,37].

Tissue sections with the implant were fixed for 5 days in 10% formalin solution, dehydrated in ethanol solutions (70%, 80%, 96% and 100% alcohol for 3 days each) and polymerized in molds for fixation using molds (Exact 41440-4150, Exact Apparetabau GmbH, Norderstedt, Germany). A light-curing resin (Technovit 7200 VLC, Sulzer, Wintertur, Swiss) was used using the polymerization unit (Exact 520–530, Exact Apparatebau GmbH, Norderstedt, Germany). The polymerization process was carried out with yellow and blue light irradiation for 12 h. The implants were embedded in a polymerization unit.

The samples in the polymeric blocks were cut (Accutom 50, Struers, Cleveland, OH, USA) midaxially in a buccolingual plane into 200 μm thick sections; and cut by the cut-grind technique to obtain a final polished 50 μm thick section. Subsequently, they were stained with toluidine blue (Toluidine Blue O, Fisher Scientific, Hampton, NH, USA) for 20 min. For histopathological and histometric study, a digital camera system (DP12, Olympus, Tokyo, Japan) coupled to a light microscope (BX51, Olympus, Tokyo, Japan) and image analysis software (MicroImage 4.0, Olympus, Tokyo, Japan) were used. X80 images were taken in all regions with special interest in the lower part of the implant neck. Global histomorphometry was carried out using a custom-made program based on an image processing system (Quantimet 500 MC, Leica, Cambridge, UK). The level of osseointegration of the dental implants was determined by the percentage of bone-to-implant contact (BIC) along the entire length covered by the images. In addition, from the histologies, the bone ingrowth into the threads (BAT) and the bone density was calculated 1 mm outside the implant threads (ROI). More than 1000 samples were analyzed with the optical microscope [30,31].

### 2.6. Statistical Analysis

Statistically significant differences among test groups studied were evaluated using statistical software (MinitabTM 13.1, Minitab Inc., Philadelphia, PA, USA). ANOVA tables with multiple comparison Fisher test were calculated. The level of significance was established at p-value<0.05.

## 3. Results

**Figure 3:**
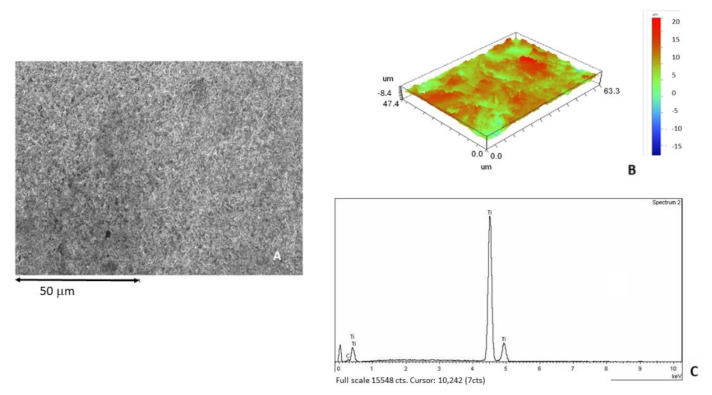
(**A**) Surface of the as-received sample observed by SEM. (**B**) Roughness in 3D image. (**C**) X-ray microanalysis.

Figure 3A shows the dental implant surface as-received without sandblasting treatment. Figure 3B shows the roughness in 3D by interferometric microscope and Figure 3C shows the X-ray microanalysis, where only titanium was detected.

**Figure 4:**
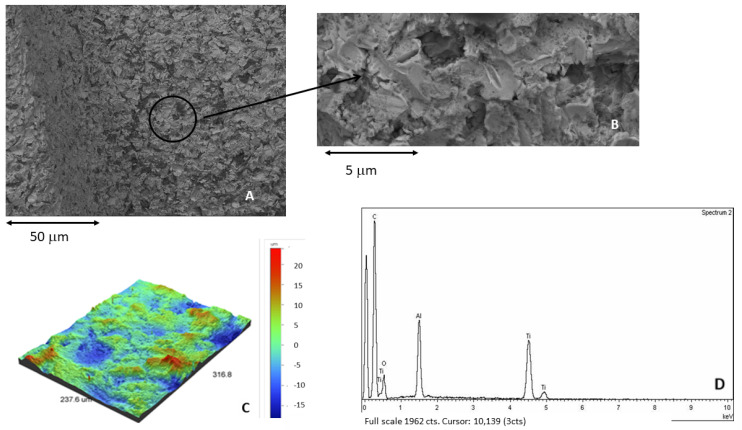
(**A**) Surface of the samples treated by sand blasting using alumina as abrasive. (**B**) At higher magnification. Presence of residual alumina on the surface. (**C**) Roughness in 3D image. (**D**) X-ray microanalysis showing the presence of aluminum.

Figure 4A corresponds to the shot blasted titanium containing residual Al_2_O_3_, showing the roughness of the titanium. At higher magnifications, the presence of alumina particles on the titanium surface can be observed (Figure 4B). Figure 4C showed the 3D roughness by interferometric microscope, also showing the presence of aluminum by the X-ray microanalysis (Figure 4D).

**Figure 5:**
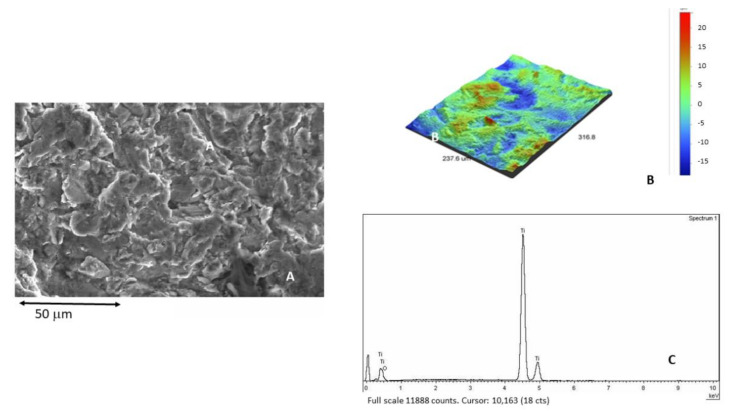
(**A**) Titanium surface after the cleaning process. (**B**) 3D interferometric image of titanium surface without alumina particles. (**C**) X-ray microanalysis not showing the presence of aluminum.

After the severe cleaning process, the roughened surface without alumina particles can be observed (Figure 5A). Figure 5B reveals the surface of titanium interferometric 3D images. Figure 5C is an example of the twenty EDX microanalysis where the aluminum is not detected.

The values of the roughness parameters obtained are shown in Table 1. The differences obtained for the roughness parameters S_a_, S_m_ and index area confirmed no statistically significant differences (*p* < 0.05) between the samples with residual alumina and the absence of the residual abrasives.

In order to ensure the surface cleanliness of the dental implants, 20 EDS microanalysis was performed on different areas of the surfaces to observe the X-ray diffractograms for each sample. When none of them showed peaks corresponding to aluminum, the samples were considered clean. From the results of microanalysis, the samples contaminated with residual alumina on the surface presented around 9.23% ± 6.22%. For the samples cleaned, the presence of aluminum was lower than the sensibility of the EDS microanalysis, 0.80%.

The water contact angles (CA), and the calculated values for the surface free energy (SFE) and its compounds following the Owens and Wendt approach are shown in Table 2 and Table 3 [19,29,30,31]. Overall, the grit-blasting treatment decreased surface wettability, i.e., increased CA. This effect was particularly pronounced for those surfaces grit-blasted with residual particles of Al_2_O_3_.

When comparing dispersive or polar components of SFE, there was a general trend in the polar component to decrease when the samples contained the presence of alumina (Table 3). Statistically significant differences in the polar component of rough surfaces with alumina with respect to control and clean surfaces were determined for the samples treated with the largest particles.

The values of colonies forming unities (CFU’s) per square millimeter (*p* < 0.005) are shown in Table 4, both strains showed a lower trend to attach on control than on rougher surfaces [38]. However, on rougher surfaces with Al_2_O_3_, results showed less bacteria attached in both strains compared to the clean samples. In the case of *L. salivarius,* the number of bacteria on all kinds of surfaces was significantly higher compared with *S. sanguinis*. Furthermore *L. salivarius* showed the same arrangement on all surfaces, showing clusters and short chains.

As the Al_2_O_3_ and clean samples presented the same roughness, these results showed the possible trend to bacteria adhesion on surfaces correlated to particles chemical composition because in general the CFU´s/mm^2^ was lower on Al_2_O_3_ shot-blasted compared to control and clean surfaces.

**Figure 6:**
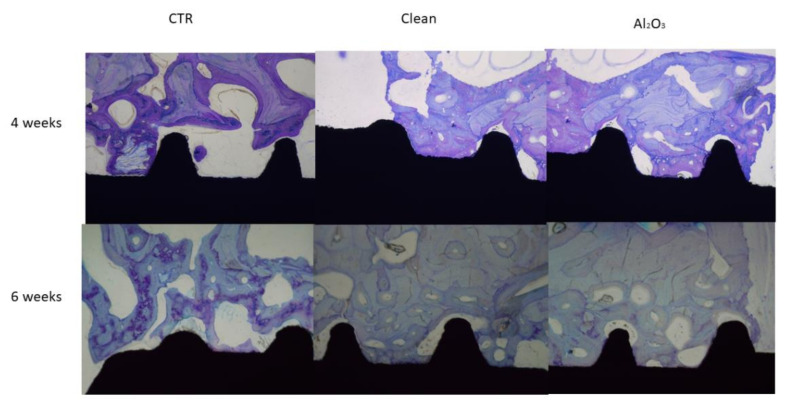
Histologies of the dental implants after 4 and 6 weeks of implantation for the three surfaces studied.

Increased roughness (Al_2_O_3_ and clean) produced a greater amount of bone and BIC. These results demonstrate that roughness results in an increased ability to attract osteoblasts to initiate bone remodeling (Table 5) In Table 6 and Table 7 are shown the BAT and ROI of the differebr dental implants after 4 and 6 weeks of implantation. From the images the results were quantified by histomorphometric analysis, showing significantly higher results for the Al_2_O_3_ samples after 4 and 6 weeks of implantation (Figure 6) [39].

In vivo tests are very important in providing information about biological reaction to implants. Histological studies demonstrate that the presence of residual blasting of Al_2_O_3_ favors the osseointegration of titanium dental implants [40].

Even as referred to in other articles, residual alumina particles cause an increase in osteoblastic adhesion, proliferation and differentiation [41,42] due to their negative charge, which causes a certain selective protein adsorption, especially of fibronectin that favors osteoblastic migration to the surface. These results and the wide clinical application of titanium shot-blasted dental implants with Al_2_O_3_ demonstrate the fallacy that debris can negatively affect osseointegration. From the ROI and BAT studies it can be seen that in both cases the dental implants with alumina have a higher amount of bone. What is remarkable is that the bone is mature and well formed.

## 4. Discussion

Abrasive blasting increases the roughness and decreases surface wettability and the total surface energy, increasing the reactivity [29]. This effect was more pronounced for surfaces with residual alumina. It is well known that surface properties influence cell interaction, orientation, migration, growth and differentiation of adherent cells. This interaction is strongly influenced by the organization and adsorption of surface-associated adhesive proteins.

It is important that the surface of an implant has a hydrophilic surface, which improves cellular anchorage and vascularization in the area, since the level of vascularization of the peri-implant tissues in the area of contact with the tissues is very poor, thus improving healing and reducing inflammation of these tissues [29,30,43,44,45,46].

In our study, the contact angle values in water were 75.4° for the alumina surface and 66.8° for the clean surface. According to the usual classification, both surfaces are considered weakly hydrophilic, as the contact angle is less than 90° [29,31,43,44].

The surface free energy of a solid is constituted by the addition of its polar and dispersive components. The higher the non-polar or dispersive character of a surface, the more difficult adhesion is in general because it depends on the liquid on the surface. The opposite is true for a surface of a more polar character, which reflects hydrophilic interactions [31]. In our study, no statistically significant differences were observed in the polar component between the alumina and clean groups. Both types of surface samples presented indentations and craters, giving rise to heterogeneous surfaces with concavities that could have influenced, decreasing wettability and surface energy in relation to the control surface.

The bacterial adhesion on titanium surfaces presents different stages: the migration of the bacteria to the surface, the adhesion, the attachment and the colonization in order to produce a biofilm. One of the most important factors to influence bacterial adhesion is the surface roughness and morphology. In addition, other factors are the hydrophobicity and surface free energy [30,31].

Roughness will provoke plaque formation and maturation, and high-energy surfaces will collect more bacteria, will bind the plaque more strongly and will select specific bacteria. Although both variables interact with each other, the influence of surface roughness overrules that of the surface free energy. From the results of our study (Table 4), the samples treated with sand blasting presented a higher bacteria adhesion than the control, showing the key role of the roughness on the microbiological behavior. Furthermore, from these results, it can be observed that the samples with residual alumina showed lower bacterial adhesion in both (*S. Sanguinis* and *L. Salivarius*) than the clean implants. This fact is due to the decrease in the surface energy produced by the alumina on the titanium surfaces in relation to clean surfaces [30,33,47].

The use of alumina has been controversial because some authors comment on the potential risk of the presence of residual blasting particles, with dissolution of aluminum ions into the host tissue not being able to be excluded. This fact has not been demonstrated and is only a comment in their contributions. The authors reported that the aluminum ions may inhibit normal differentiation of bone narrow stromal cells and normal bone deposition and mineralization [36,48]. Besides, aluminum has been shown to induce net calcium efflux from culture bone [48]. It is well known that the aluminum oxide (alumina) is considered bioinert and is insoluble. The ion release of aluminum from alumina immersed in simulated body fluid (SBF) is much lower than Ti-6Al-4V [49,50,51].

In addition, the studies that are often referenced are on femoral hip balls made of alumina. This orthopedic application is very different from that of dental implants, since the femoral ball is made entirely of alumina and in our dental implants the surface content is approximately 8%. Furthermore, the mechanical stresses and wear conditions in hip prostheses are not comparable with dental implants [11,52,53]. Therefore, the comparisons are not appropriate.

Some contribution explains that the aluminum may compete with calcium in the healing implant bed, and aluminum can accumulate at the mineralization front and in the osteoid matrix itself [5]. Nimb et al. [54] found that aluminum inhibited the formation of calcium phosphate crystal and the hydroxyapatite crystalize poorly in a study in Beagle dogs. However, Quarles et al. [55] demonstrated that the aluminum stimulated bone formation with an important increase in trabecular bone volume. In this sense, the study of Lau et al. [42] also showed that the presence of aluminum ions favors the bone formation in an in vitro study. It is important to note that some studies do not work with the presence of alumina but with aluminum ions, and this fact can give completely opposite results [56,57,58]. It is well known that compounds of a metal have very different properties from the pure metal and its ions [53].

Several researchers, such as Feighan et al. [59] and Piatelli et al. [26] studied the biological behavior with Al_2_O_3_. In the first, they showed that the blasted implants presented woven and lamellar bone in direct apposition to the implant surface, and this fact was demonstrated in the active bone formation towards the implants. In the second research, the histological results did not provide evidence to support that the residual aluminum oxide particles on the dental implant affect osseointegration. Besides, very low levels of trace aluminum have been found in various organs and in blood when alumina was used [60]. Piatelli et al. [26] in their interesting study indicated that there is no significant effect of alumina on osseointegration. However, this work did not determine the effectiveness of the decontamination process of dental implants, nor did it determine the residual alumina content or parameters, such as roughness, contact angle and other surface properties. In addition, the rabbit animal model, having virtually no trabecular bone, may affect the histology results. The uniqueness of our study is that all dental implants have been sand blasted in the same way and under the same conditions. Consequently, all dental implants have the same roughness and the same surface energy. Cleaning treatments on a batch of dental implants after sand blasting removes the alumina residues but does not affect the topography of the implant. Having some implants treated with sand blasting and others not gives rise to differences in surface roughness and surface energy that result in different behaviors. This work complements this study.

As reported in Feighan’s [59] and Piatelli’s [26] publications, in our in vivo study, any toxic effect due to alumina nor inflammation reaction differences between the clean or alumina surfaces were reported.

In our study, statistically significant differences were found between the implants with residual alumina and the cleaned implants regarding bone-implant contact. This fact is due to the effect of alumina on the wettability and surface energy, which favors the osteoblast activity. It should be noticed that this new bone tissue is continuous with the lamellar bone filling the threads of the implant outer area (ROI). The new formed tissue held a high level of maturity in all evaluated regions with a general prevalence of lamellar tissue with some osteons and a rare presence of woven bone tissue. These results are in contrast with those found with Ti-6Al-4V alloy implants, where differences in bone-implant contact percentages or removal torques values were presented by Wennenberg et al. The roughness of these samples were different between samples [37,60]. These authors confirmed that the detected amounts of Al are much higher on Ti-6Al-4V and the ion release level of the alumina were traces lower than 5 ppb for 1 month [61]. It is well known that aluminum oxide is very stable with a negligible solubility index, with this oxide considered insoluble and therefore the action of aluminum ions is practically negligible [49,51].

On the other hand, Esposito et al. [62] found that residual abrasive particles are not the cause of the failures or fractures of the dental implants. The fractures are due to the positive residual stresses (tensile) on the surface produced by a deficient sand blasting process. Aparicio et al. [63] also demonstrated that the dental implants with residual alumina did not present a higher corrosion rate in relation to the clean implants due to the bioinert character of the alumina. However, when SiC is used as an abrasive, this carbide produces an increase in the corrosion rate due to SiC can be oxidized to SiO_2_ [63].

## 5. Limitations of the Study

The study demonstrates that residual alumina improves osseointegration in titanium with the same roughness. However, X-ray energy dispersive microanalysis analysis has a sensitivity of 0.8% and more sensitive techniques could be used to quantify more accurately the percentage of residues. Furthermore, this bacterial study is on adhesion, but biofilm studies are necessary to be able to conclude in a more rigorous way the bactericidal capacity of the surface with residual alumina. Further studies with different bacterial strains are needed to determine the mechanism of the possible bactericidal effect.

## 6. Conclusions

It can be stated that roughness is the most important surface parameter for osteoblast adhesion, osseointegration as well as for the colonization of microorganisms. It has been also demonstrated that alumina residues used as abrasives (approx. 8%) in titanium implants result in a more hydrophobic surface with a lower surface energy than the dental implants without alumina residual. These physico-chemical parameters favor osteoblast cell adhesion. The lower surface energy and the polarity of the alumina reduced the colonization of the bacteria studied (*L. Salivarius* and *S. Sanguinis*) compared to dental implants with the same surface roughness but without the presence of alumina. In vivo tests showed a statistically higher level of bone implant contact (BIC) than cleaned implants. Further studies will be needed to determine the effects of wettability and surface energy on osteoblastic activity, and also the possible bactericidal effect of alumina. Further studies will undoubtedly be necessary to confirm the bactericidal effect of titanium and to determine the mechanisms of this behavior.

## Figures and Tables

**Table 1:** Roughness of the titanium surface. Statistical differences for each column are indicated by single asterisk and double asterisk symbols (*p* < 0.05).

	Sa (µm) ± SD	Sm (µm) ± SD	Index Area ± SD
Ctr	0.21 ± 0.02 *	0.34 ± 0.02 *	1.09 ± 0.01 *
Al_2_O_3_	2.35 ± 0.13 **	5.41 ± 0.21 **	1.18 ± 0.06 **
Clean	2.34 ± 0.25 **	5.67 ± 1.07 **	1.16 ± 0.04 **

**Table 2:** Apparent contact angles for the three liquids used on the different c.p. Ti surfaces. Values are mean ± standard deviation. Statistical differences vs. smooth surfaces for each column are indicated by single and double or triple asterisk symbols (*p* < 0.05).

Surface	Water CA’ [°]	Di-Iodomethane CA’ [°]	Formamide CA’ [°]
Ctr	66.3 ± 0.5 *	51.5 ± 0.9 *	51.8 ± 1.0 *
Alumina	75.4 ± 0.5 **	62.2 ± 1.2 **	59.3 ± 2.0 **
Clean	66.8 ± 0.7 *	38.5 ± 1.4 ***	35.0 ± 1.7 ***

**Table 3:** Surface energy and its components for the different Ti surfaces. Values are mean ± standard deviation. Statistical differences for each column are indicated by single asterisk and double asterisk symbols (*p* < 0.05).

Surface	Surface Energy (mJ/m^2^)		
	Total	Dispersive Component	Polar Component
Ctr	40.0 ± 3.5 *	24.8 ± 3.2 *	15.2 ± 4.0 *
Alumina	28.2 ± 1.9 **	17.7 ± 1.1 **	10.5 ± 3.1 **
Clean	38.8 ± 2.5 *	26.8 ± 2.6 *	11.0 ± 3.4 **

**Table 4:** CFU’s quantification for different titanium surfaces. Statistical differences are expressed by a single asterisk for each column (*p* < 0.05).

Material	S. Sanguinis CFU’s	L. Salivarius CFU’s
Control	4.09 × 10^1^ ± 8/mm^2^	6.21 × 10^3^ ± 4.75 × 10^2^/mm^2^
Al_2_O_3_	4.94 × 10^1^ ± 12/mm^2^	6.93 × 10^3^ ± 6.45 × 10^2^/mm^2^
clean	9.73 × 10^1^ ± 9/mm^2^ *	8.09 × 10^3^ ± 4.88 × 10^2^/mm^2^ *

**Table 5:** Bone index contact (BIC) for different types of Ti dental implant surfaces and after 4 and 6 weeks of implantation. Statistical differences for each column are indicated by single asterisk and double or triple asterisk symbols (*p* < 0.05).

Material	4 Weeks	6 Weeks
Control	22% ± 5% *	27% ± 8% *
Al_2_O_3_	49% ± 10% **	70% ± 9% **
clean	34% ± 6% ***	55% ± 8% ***

**Table 6:** Total bone area (BAT) for different types of Ti dental implant surfaces and after 4 and 6 weeks of implantation. Statistical differences for each column are indicated by single asterisk and double or triple asterisk symbols (*p* < 0.05).

Material	4 Weeks	6 Weeks
Control	16% ± 4% *	19% ± 7% *
Al_2_O_3_	42% ± 9% **	62% ± 8% **
clean	29% ± 6% ***	45% ± 5% ***

**Table 7:** Region of interest (ROI) bone growth for different types of Ti dental implant surfaces and after 4 and 6 weeks of implantation. Statistical differences for each column are indicated by single asterisk and double or triple asterisk symbols (*p* < 0.05).

Material	4 Weeks	6 Weeks
Control	21% ± 7% *	29% ± 6% *
Al_2_O_3_	44% ± 6% **	68% ± 8% **
clean	32% ± 5% ***	50% ± 7% ***

## Data Availability

The authors can provide details of the research requesting by letter and commenting on their needs.
